# First experience of lymphaticovenular anastomosis using BHC RobotiScope: A case report

**DOI:** 10.1097/MD.0000000000033841

**Published:** 2023-05-17

**Authors:** Jae-Ho Chung, Dong-Jin Kim, Eul-Sik Yoon, Seung-Ha Park

**Affiliations:** a Department of Plastic and Reconstructive Surgery, Korea University Hospital, Seoul, Republic of Korea; b Institute of Nano, Regeneration, Reconstruction, College of Medicine, Korea University, Seoul, Republic of Korea.

**Keywords:** lymphaticovenular anastomosis, lymphedema, robotic surgery

## Abstract

The RoboticScope (BHS Technologies GmbH, Innsbruck, Austria) is a robotic exoscope, which consists of a robotic arm that holds a 3-dimensional camera. It has an advantage that a surgeon can perform an operation comfortably with a favorable ergonomic position. Also, it allows the delivery of clear and high-quality visualization for surgeons. In this study, we would like to share our initial experience with this newly developed microscope technology in lymphaticovenular anastomosis (LVA). To the best of our knowledge, it is the first experience of LVA using this microscope in Asia.

**Patient concerns:**

A 65-year-old woman presented with bilateral lower extremity lymphedema after a hysterectomy that was performed 25 years back. Despite complex decongestive physiotherapy, an edematous symptom in both legs worsened.

**Diagnoses:**

In lymphoscintigraphy, a decreased visualization of main lymphatic flow in both the lower extremities was evident which was further suggestive of lymphatic obstruction.

**Intervention:**

Although both sides showed edematous symptoms, we decided to proceed with the surgery on the left side first, because of the worsened condition. Four LVAs were performed at the dorsum of the foot (×2), ankle, and the superior edge of the knee using RoboticScope

**Outcomes:**

At 6-months follow-up after operation, the postoperative circumference diameters were improved than preoperative in 10 cm above the knee (45 cm vs 49 cm), 10cm below the knee (37 cm vs 41 cm) and lateral malleolus (25 cm vs 28 cm). The lower extremity lymphedema index was also improved from 346.7 to 287.4 postoperatively. The RoboticScope provided a high-resolution image and a favorable ergonomic position during an operation.

**Lessons:**

The results represent the possibility of the application of a robotic microscope in the field of microsurgery, and further studies are necessitated to confirm the efficacy of this system.

## 1. Introduction

In recent times, physiologic surgery including lymphaticovenular anastomosis (LVA) has become the mainstay of treatment for patients with lymphedema. In particular, LVA has been widely performed around the world since 2000, when the concept of super micro surgery for anastomosis of small vessels measuring <0.8 mm was introduced, and the performance of surgical microscopes has continued to improve along with advances in microsurgery.

To achieve a successful LVA, the following conditions are necessary. First, a high magnification of more than 25 to 30 times is required to perform precise sutures between the lymphatic vessel and venule with a diameter of <1 mm. Second, since multiple anastomoses are performed for a long time on 1 side of the upper or lower extremity of the patient, it is important to secure the optimal surgical field of vision and ergonomic position for the operator regardless of the patient’s posture or the location of anastomosis.^[1]^ In this respect, the performance of the microscope, as well as the microsurgical skill of the operator, are very important for the accuracy and safety of the operation.

It has been more than 20 years since robotic surgery was actively performed and there have been immense efforts to use this system in the field of microsurgery. In particular, new types of microscopes such as MUSA, Symani, and Aeos have been developed subsequently. In the current publication, we would like to share our experience of treating a patient with lymphedema by performing LVA using RoboticScope by BHS Technologies (Innsbruck, Austria). To the best of our knowledge, this is the first case of LVA using RoboticScope reported in Asia, and we expect it to be a reference for the vast application of robotic microscopes in the future.

## 2. Case description

**Figure 1.**
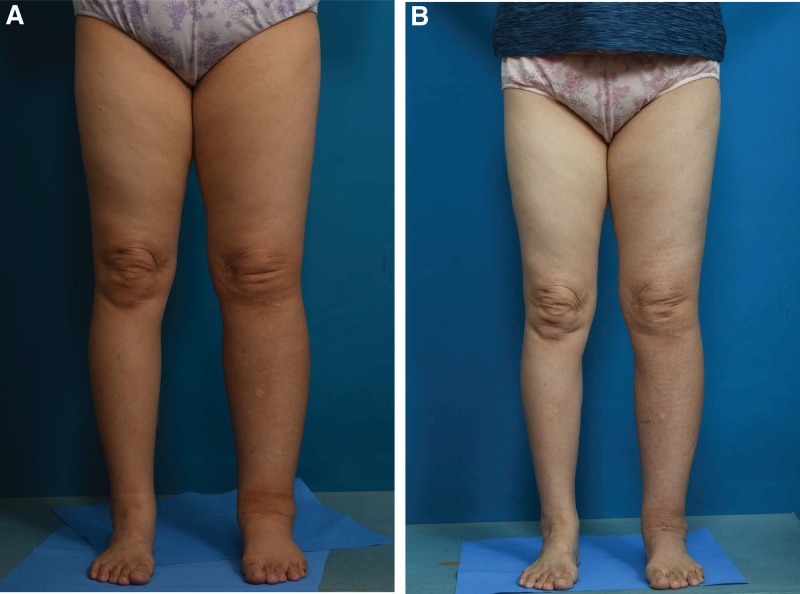
Clinical photograph of the patient. (A) A 65-year-old woman manifested bilateral lower extremity swelling after a 25 years history of hysterectomy. (B) At 6 months, her left LEL index was reduced to 287.4. LEL = lower extremity lymphedema.

A 65-year-old woman presented with bilateral lower extremity lymphedema after a hysterectomy that was performed 25 years back. (Fig. 1A) In lymphoscintigraphy, a decreased visualization of main lymphatic flow in both the lower extremities was evident which was further suggestive of lymphatic obstruction. Although both sides showed edematous symptoms, we decided to proceed with the surgery on the left side first, because of the worsened condition.

**Figure 2.**
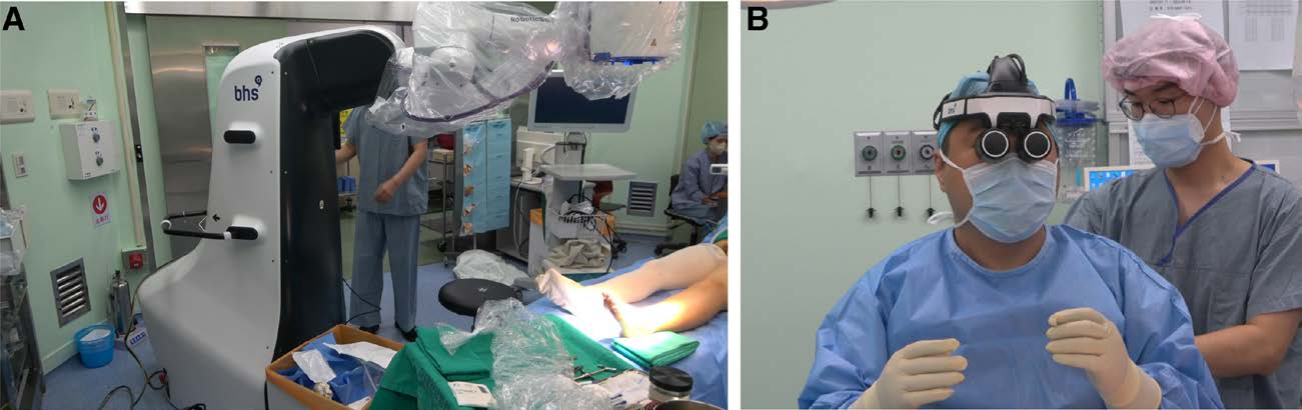
The setting of BHS Robotiscope in the operating room, (A) robotic exoscope consisting of a 6-axis robotic arm and a high-resolution 3-dimensional camera, (B) surgeon wearing a head mount display (HMD) with a motion sensor.

After intradermal injection of 0.2 cc of indocyanine green (ICG, Dongindang Pharm, Sigheung, Korea) into each webspace of the toes, the course of the lymphatic vessel was identified with a hand- held near-infrared camera (Fluobeam; Fluoptics, France). After setting up the equipment, a total of 4 LVAs were performed at the dorsum of the foot, ankle and knee. (Fig. 2) At each site, a 3-cm-long incision was usually made and the largest lymphatic vessel under the superficial fascia in the subcutaneous tissue was selected for the LVA. All LVAs were performed with 11 to 0 nylon in the end-to-end anastomosis fashion. (Supplemental Digital Content, See Video, http://links.lww.com/MD/J17) After all procedures, the patency of the anastomosis was confirmed using the above-mentioned near-infrared cameras.

In this case, the preoperative circumference diameters were 49 cm and 41 cm at 10 cm above and below the patella, and 28 cm at the lateral malleolus. The postoperative circumference diameters were improved to 45 cm, 37 cm and 25 cm at 6 months, respectively. The lower extremity lymphedema index also improved from 346.7 to 287.4 postoperatively. (Fig. 1B) It showed a pattern of reducing edema, and there were no complications.

## 3. Discussion

RoboticScope was originally introduced by targeting the fields of otology or neurosurgery, but it is now expanding its scope to the field of super micro surgery.^[2–5]^ A case report of LVA with this scope has already been published in 2021.^[1]^ However, this scope has not yet been officially launched in the Asian market, and demonstrations have been underway at several hospitals since 2022.

RoboticScope consists of a body with a high-resolution 3-dimensional camera, a 6-axis robotic arm, an external touch screen, and a head mount display (HMD) with a motion sensor worn by a surgeon. The most important optic performance is to achieve a magnification field of up to 30.1X at a working distance of 300 to 600 mm. The surgeon can see the menu screen consisting of inner and outer circles through the eyepieces of HMD. By moving the head in the desired direction, the surgeon can switch between the 2-dimensional and 3-dimensional image, magnification adjustment, and light intensity of the image while leaving hands on the surgical field.^[6]^ If necessary, it can be configured to provide an actual visual field by raising the eyepiece of HMD. All these features can provide an ideal ergonomic posture for a microsurgeon.

A remarkable advantage of RoboticScope is that a surgeon can perform LVA comfortably with a favorable ergonomic position in areas where maintaining a posture is difficult, such as a superior edge of the knee point on the knee.^[7]^ Khansa et al^[8]^ reported that 78.3% of plastic surgeons underwent musculoskeletal symptoms, commonly in the neck, shoulders, and lower back. In particular, these symptoms were most triggered after microscope use. (OR 1.64, *P* < .01). Therefore, for the longevity of the microsurgeon and surgical efficiency, this scope is expected to be a good alternative.

The second advantage of RoboticScope is that it allows the delivery of clear and high-quality visualization for surgeons. In particular, it was possible to confirm the lymph flow and the resulting movement of red blood cells without blurring even in the maximum field of vision, so it provides immense help in the evaluation of patency after anastomosis. Lastly, under the instruction of a company official, no problems were encountered while performing LVA with just 1 to 2 hours of simple practice. A surgeon skilled in microsurgical techniques could easily get adapted to this system without a special learning curve.^[9]^ Compared to other equipment using a robotic hand with a controller such as Symani, it was possible to anastomose naturally while feeling the tactile feedback.

However, as the technique is still in its early form, there are some limitations to this equipment. First, there were often moments when the screen was blurred because the autofocusing function did not work. Second, it was necessary to adapt to the 3-dimensional image because the depth was expressed only in 3-dimensional screen mode in inclined areas such as superior edge of the knee point. Third, because the fluorescent filter which is the most important part of lymphatic surgery was absent, the patency after anastomosis had to be evaluated additionally through other near-infrared cameras. Nevertheless, the considerably high prices and operating costs are thought to be the biggest obstacle to the supply of this equipment.^[9]^ Compared to conventional microscopes, it is not yet familiar, and there is an aspect where price competitiveness is somewhat lower at this time. However, just as smartphones replaced everyday life, this type of exoscope system is expected to change significantly with the development of technology. Since the manufacturing company is currently improving both hardware and software with feedback from many surgeons, the instrument is anticipated to become a practical alternative in the future.

In summary, this case represents the possible application of a robotic microscope in the field of microsurgery, even in super micro surgery. The outcome of the treated patient was good and there were no complications. Intraoperatively, it provided an ergonomic working position and good visualization for a surgeon. In the future, further evidence will confirm the efficacy of this new technology, which could help improve surgical outcomes.

## Author contributions

**Conceptualization:** Jae-Ho Chung.

**Data curation:** Jae-Ho Chung, Dong-Jin Kim.

**Writing – original draft:** Jae-Ho Chung.

**Writing – review & editing:** Eul-Sik Yoon, Seung-Ha Park.

## Supplementary Material
